# Benign cervical schwannoma with tracheal invasion

**DOI:** 10.31744/einstein_journal/2024RC0528

**Published:** 2024-01-29

**Authors:** Leonardo Lima Lorandi, Fabio Pupo Ceccon, Fabiano Mesquita Callegari, Carlos Eduardo Bacchi, Leonardo Haddad

**Affiliations:** 1 Faculdade Israelita de Ciências da Saúde Albert Einstein Hospital Israelita Albert Einstein São Paulo SP Brazil Faculdade Israelita de Ciências da Saúde Albert Einstein , Hospital Israelita Albert Einstein , São Paulo , SP , Brazil .; 2 Hospital Israelita Albert Einstein São Paulo SP Brazil Hospital Israelita Albert Einstein , São Paulo , SP , Brazil .; 3 Pathology Department Universidade Federal de São Paulo São Paulo SP Brazil Pathology Department , Universidade Federal de São Paulo , São Paulo , SP , Brazil .; 4 Laboratorio de Patologia Bacchi Ltda Botucatu SP Brazil Laboratorio de Patologia Bacchi Ltda , Botucatu , SP , Brazil .; 5 Department of Otorhinolaryngology and Head and Neck Surgery Escola Paulista de Medicina Universidade Federal de São Paulo São Paulo SP Brazil Department of Otorhinolaryngology and Head and Neck Surgery , Escola Paulista de Medicina , Universidade Federal de São Paulo , São Paulo , SP , Brazil .

**Keywords:** Neurilemmoma, Neoplasm invasiveness, Trachea, Tracheal neoplasms, Thyroid neoplasms, Thyroidectomy, Differential, diagnosis

## Abstract

Schwannomas commonly develop in the cervical region, 25% – 45% of cases are diagnosed in this anatomical region. Tracheal neurogenic tumors are exceedingly rare and can be misdiagnosed as invasive thyroid carcinomas or other infiltrating malignancies when present at the level of the thyroid gland. Here, we present a case of synchronous benign cervical schwannoma with tracheal invasion and papillary thyroid carcinoma in a patient who was initially hospitalized for COVID-19. The patient presented with dyspnea that was later found to be caused by tracheal extension of a cervical tumor. Surgical excision was performed, and the surgical team proceeded with segmental tracheal resection, removal of the cervical mass, and total thyroidectomy. The specimen was sent for pathological analysis, which revealed synchronous findings of a benign cervical schwannoma with tracheal invasion and papillary thyroid carcinoma. The literature on this subject, together with the present case report, suggests that neurogenic tumors should be included in the differential diagnosis of obstructing tracheal cervical masses. Surgical excision is the first-line of treatment for benign cervical schwannomas.

## INTRODUCTION

Schwannomas are benign nerve sheath tumors composed of Schwann cells that are found in most peripheral and cranial nerves, except for the olfactory, optic, and autonomic nervous systems. ^( 1 , 2 )^ Primary tracheal tumors are rare oncological entities accounting for approximately 1% of all cancer cases. ^( 3 )^ Schwannomas of the head and neck anatomic site account for 25% – 45% of the burden of this disease, although they account for only 0.5% of all primary tracheal tumors. ^( 1 )^ Tracheal schwannomas most commonly affect the distal airways and are found in the bronchi and lung parenchyma. ^( 4 , 5 )^

## CASE REPORT

**Figure 1:**
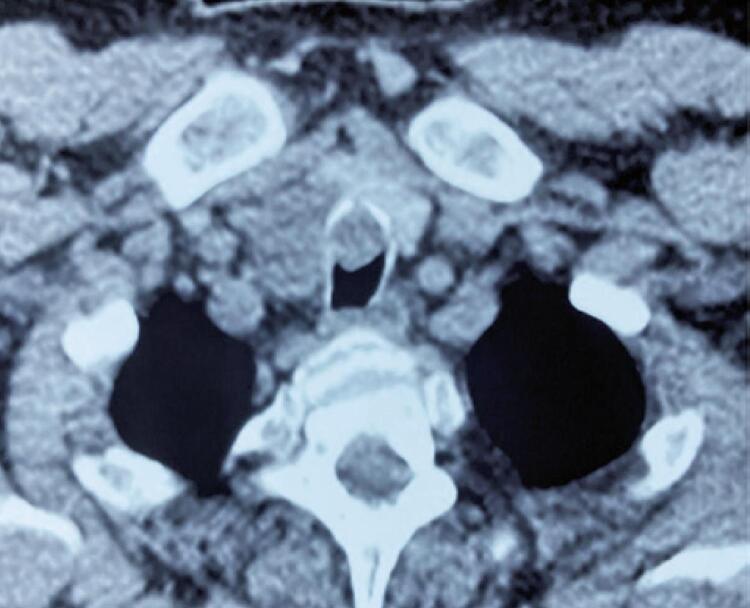
Computed tomography of the neck with contrast showing evidencing cervical tumor with extension to tracheal lumen

**Figure 2:**
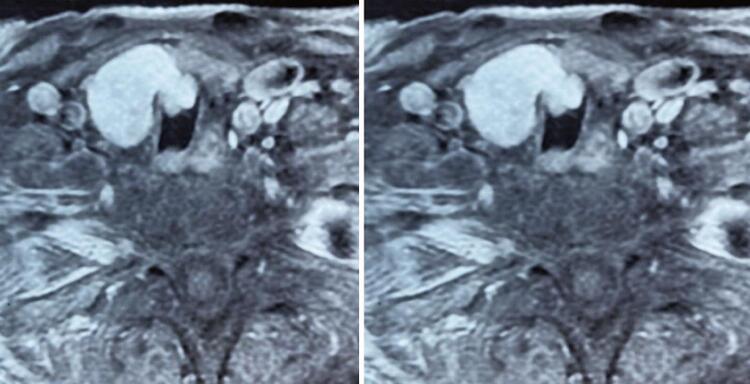
Contrast-enhanced magnetic resonance imaging showing cervical tumor extending to the tracheal lumen

**Figure 3:**
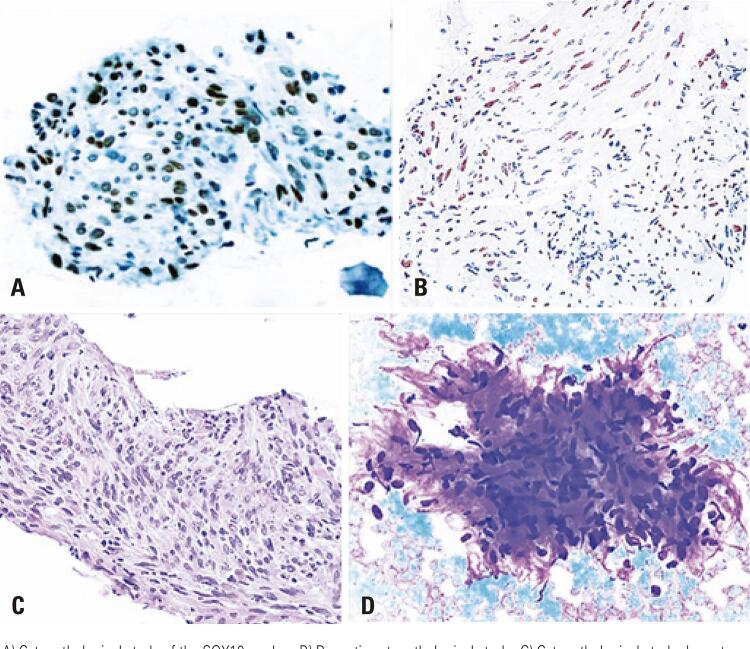
Cytopathological and immunohistochemical findings of fine-needle aspiration biopsy A to D

**Figure 4:**
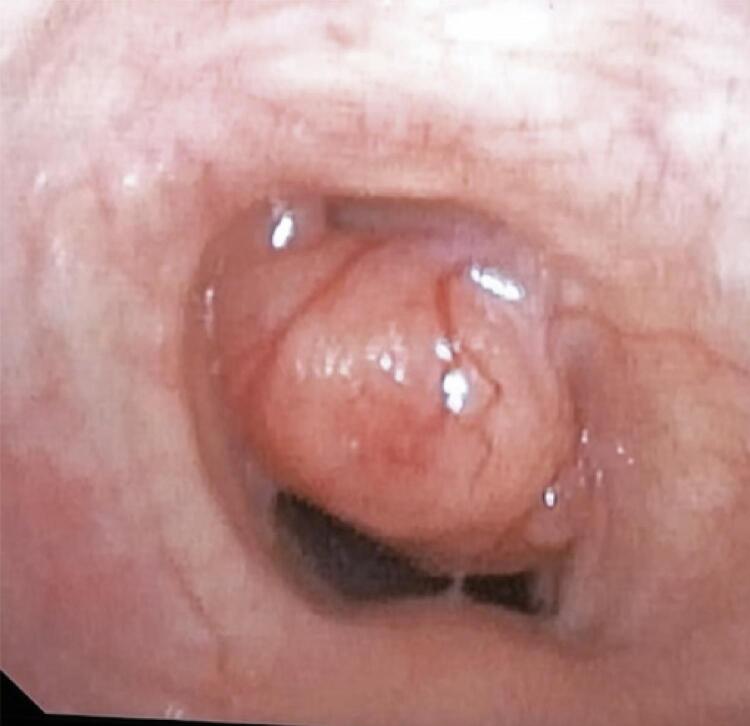
Endoscopic vision of the trachea: partially obstructing lesion occupying 80% of the tracheal light at the level of the third tracheal ring

**Figure 5:**
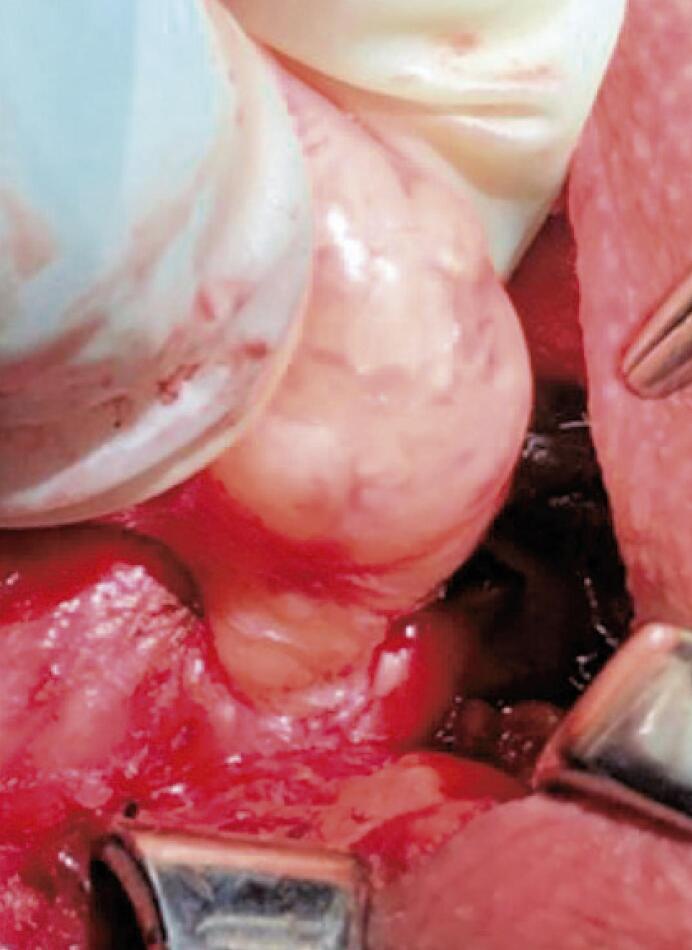
Intraoperative image without involvement of the recurrent laryngeal nerve or vagus nerve and extension of the tumor to the tracheal lumen between the 3rd and 4th tracheal rings

**Figure 6:**
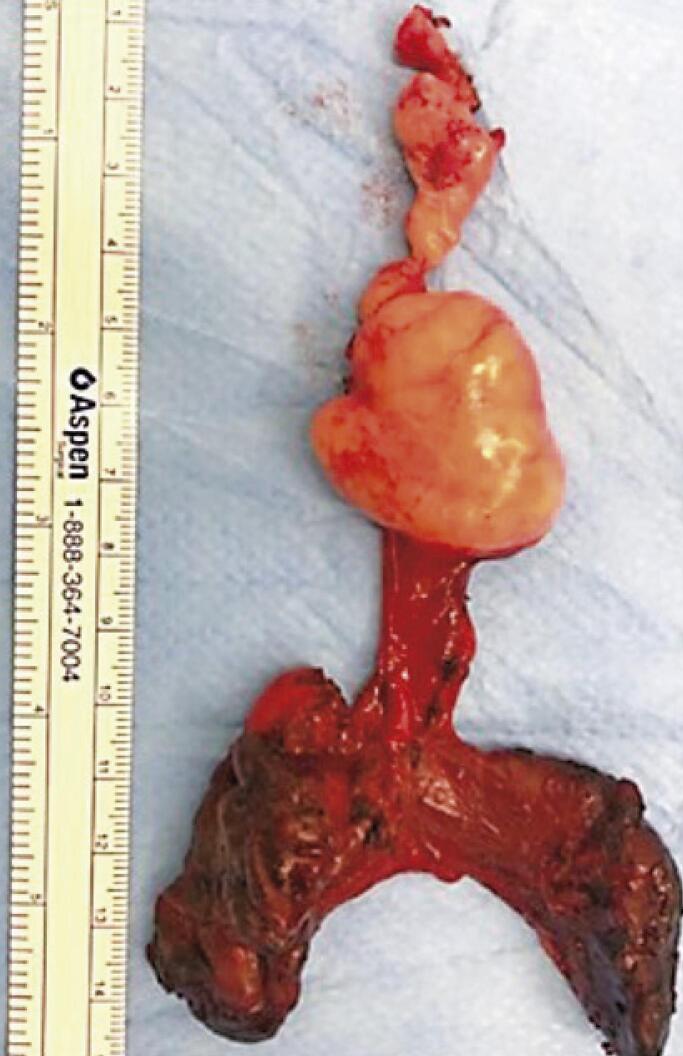
Surgical specimen: resection of the cervical tumor with intratracheal extension and en bloc thyroid gland

A 61-year-old man with no significant medical history had been recently diagnosed with COVID-19 inflammatory lung disease (ILD). In the prior three months, he had developed persistent respiratory discomfort. At that time, chest computed tomography was performed to evaluate the extent of lung disease ( Figure 1 ), which showed mild ILD, but a cervical mass with tracheal ingrowth vegetation was found. The workup included neck magnetic resonance imaging (MRI), which revealed a solid thyroid nodule in the middle third of the right inferior lobe with a high-intensity signal on T2-weighted images, a hypointense mass on T1-weighted images, and no restricted diffusion. With contrast infusion, the mass showed marked enhancement and was measured at 3,9 X 3,7 X 3,7cm. The lesion also had an area of tracheal involvement that was assessed at 1,6 X 1,7cm and occupied two thirds of the tracheal lumen ( Figure 2 ). The main part of the thyroid nodule was biopsied using fine-needle aspiration (FNA). Pathological examination revealed atypia of undetermined significance (AUS) composed of fusiform cells. A decision was made to repeat the US-guided FNA biopsy. On ultrasound (US), two lesions were shown. The new cytology report described a left-lobe nodule suspected to be a papillary thyroid carcinoma (PTC), while the cervical mass revealed spindle cells. Immunohistochemically, these fusiform cells showed diffuse positivity for SOX10 expression and were negative for cytokeratin, Tg, CD34, and p63, a pattern suggestive of peripheral nerve sheath neoplasia ( Figure 3 ). Surgery was planned along oncological principles due to the risk of malignant disease. The patient underwent bronchoscopy on the day of surgery. Tracheoscopy revealed a lobulated mass occupying approximately 80% of the tracheal lumen ( Figure 4 ). General anesthesia was successful after placement of the endotracheal tube’s (ETT) balloon distal to the lesion. Access was obtained through transverse cervicotomy. A whitish cervical tumor was visible and had a clear dissection plane relative to the thyroid parenchyma. The vagus and recurrent laryngeal nerves were dissected carefully. Total thyroidectomy was performed. The extracapsular part of the cervical tumor was removed in an en bloc fashion by wedge resection of the tracheal mass, together with the anterior walls of the 3 ^rd^ and 4 ^th^ tracheal rings, which appeared fused upon further inspection (Figures 5 and 6). Surgical margins were evaluated with intraoperative frozen section pathology, showing no tumor involvement. Tracheal closure was accomplished using simple interrupted 3-0 polyglactin sutures. At the postoperative follow-up, the patient presented with improvement in his dyspnea without motor or sensory deficits. Tracheoscopy revealed no stenotic scarring or granuloma. After 12 months, the patient remained free of recurrence.

A) Cytopathological study of the SOX10 marker; B) Panoptic cytopathological study; C) Cytopathological study: hematoxylin-eosin staining of the cell block; D) Immunohistochemical nuclear expression of SOX 10 by neoplastic schwannoma cells.

The work was approved by the Research Ethics Committee of *Hospital Israelita Albert Einstein* (CAAE: 57108722.0.0000.0071; # 5.363.574).

## DISCUSSION

When considering the tumor characteristics of this case, two hypotheses can be drawn to explain the presence of this histological entity at this particularly uncommon site. The first and most likely hypothesis is a schwannoma of the superficial cervical plexus origin that invaded the tracheal lumen. The second, less likely, hypothesis is a tumor of primary tracheal origin that invaded the surrounding cervical tissue. Regardless of the correct etiopathology, any neck mass with an intratracheal segment presenting as new or worsening dyspnea must be considered potentially malignant. However, in this case, the pathological findings confirmed the rare occurrence of a benign schwannoma invading the tracheal lumen. Few cases of tracheal schwannomas have been reported in medical literature. A systematic review published in 2015 found 51 cases with the same histology and tumor location. ^( 5 )^ The authors of this report conducted a search of the Pubmed database from 2016 to january 2022 about partially obstruction of tracheal lumen and found 13 articles describing tracheal shwannomas. When they occur in the respiratory system, they primarily present as lung or bronchial masses and seldom transform into malignant tumors. ^( 4 , 5 )^ The clinical manifestations are often insidious and nonspecific, making the diagnosis of tracheal schwannomas challenging. Tracheal elasticity contributes to delayed diagnosis because tumors in this location infrequently show symptoms in the first stages. Wheezing, coughing, hemoptysis, dyspnea, and dysphonia are typical, albeit nonspecific, complaints in patients with intraluminal tumors. Indeed, these symptoms can be mistakenly attributed to other more common pathologies and are eventually discovered as incidental exam findings, as seen in the present case report. ^( 5 )^

## CONCLUSION

Cervical schwannomas are relatively frequent; however, tracheal involvement is rare. The presence of tracheal extension should alert physicians to the possibility of malignant neoplasia. Surgery should remove the entire tumor. Even if uncommon, schwannomas should be considered in the differential diagnosis of cervical tumors involving the tracheal lumen.
